# *N*,*N*′-Bis(2,6-diisopropyl­phen­yl)-3,6-di­methyl-1,2,4,5-tetra­zine-1,4-dicarboxamide

**DOI:** 10.1107/S1600536812019186

**Published:** 2012-05-05

**Authors:** Na-Bo Sun, Guo-Wu Rao, Li-Ling Zhang

**Affiliations:** a College of Biology and Environmental Engineering, Zhejiang Shuren University, Hangzhou 310015, People’s Republic of China; b College of Pharmaceutical Science, Zhejiang University of Technology, Hangzhou 310014, People’s Republic of China

## Abstract

In the title mol­ecule, C_30_H_42_N_6_O_2_, the amide-substituted N atoms of the tetra­zine ring deviate from the approximate plane of the four other atoms in the ring by 0.457 (3) and 0.463 (3) Å, forming a boat conformation. The two benzene rings form a dihedral angle of 47.69 (9)°. Intra­molecular N—H⋯N and weak C—H⋯O hydrogen bonds are observed.

## Related literature

For chemical reactions of 1,2,4,5-tetra­zine derivatives, see: Domingo *et al.* (2009 ▶); Lorincz *et al.* (2010 ▶). For their bio­logical activity, see: Devaraj *et al.* (2009 ▶); Eremeev *et al.* (1978 ▶, 1980 ▶); Han *et al.* (2010 ▶); Neunhoeffer (1984 ▶); Sauer, (1996 ▶). For anti­tumor activity of 1,2,4,5-tetra­zine derivatives, see: Hu *et al.* (2002 ▶, 2004 ▶); Rao & Hu (2005 ▶, 2006 ▶). For standard bond lengths, see: Allen *et al.* (1987 ▶). For the synthesis of the title compound, see: Hu *et al.* (2004 ▶); Rao *et al.* (2012 ▶); Skorianetz & Kovats (1970 ▶, 1971 ▶); Sun *et al.* (2003 ▶).
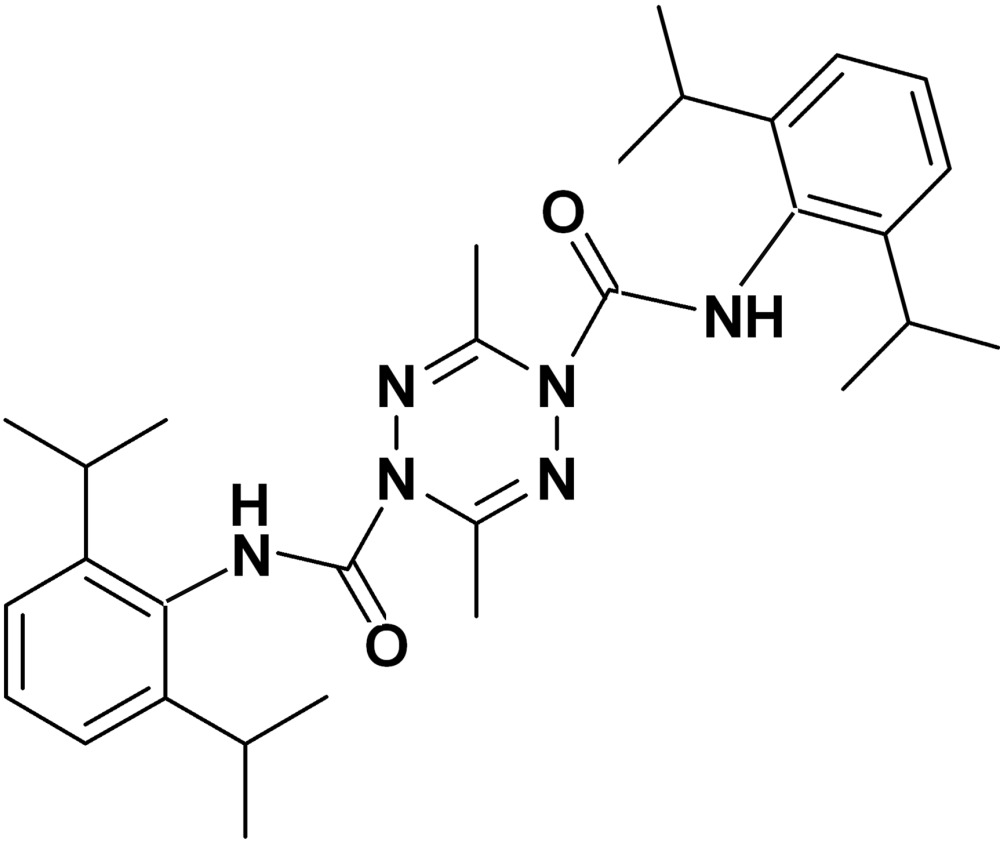


## Experimental

### 

#### Crystal data

C_30_H_42_N_6_O_2_
*M* *_r_* = 518.70
Monoclinic, 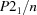

*a* = 9.0599 (14) Å
*b* = 33.203 (5) Å
*c* = 10.8082 (17) Å
β = 112.013 (2)°
*V* = 3014.3 (8) Å^3^
*Z* = 4
Mo *K*α radiation
μ = 0.07 mm^−1^
*T* = 298 K
0.38 × 0.28 × 0.20 mm

#### Data collection

Bruker SMART CCD diffractometer
Absorption correction: multi-scan (*SADABS*; Bruker, 1997 ▶) *T*_min_ = 0.973, *T*_max_ = 0.986
15158 measured reflections
5304 independent reflections
3918 reflections with *I* > 2σ(*I*)
*R*_int_ = 0.029

#### Refinement

*R*[*F* ^2^ > 2σ(*F* ^2^)] = 0.057
*wR*(*F* ^2^) = 0.154
*S* = 1.05
5304 reflections
352 parameters
H-atom parameters constrained
Δρ_max_ = 0.20 e Å^−3^
Δρ_min_ = −0.22 e Å^−3^

### 

Data collection: *SMART* (Bruker, 1997 ▶); cell refinement: *SAINT* (Bruker, 1997 ▶); data reduction: *SAINT*; program(s) used to solve structure: *SHELXS97* (Sheldrick, 2008 ▶); program(s) used to refine structure: *SHELXL97* (Sheldrick, 2008 ▶); molecular graphics: *ORTEP-3 for Windows* (Farrugia, 1997 ▶); software used to prepare material for publication: *WinGX* (Farrugia, 1999 ▶).

## Supplementary Material

Crystal structure: contains datablock(s) I, global. DOI: 10.1107/S1600536812019186/lh5460sup1.cif

Structure factors: contains datablock(s) I. DOI: 10.1107/S1600536812019186/lh5460Isup2.hkl

Supplementary material file. DOI: 10.1107/S1600536812019186/lh5460Isup3.cdx

Supplementary material file. DOI: 10.1107/S1600536812019186/lh5460Isup4.cml

Additional supplementary materials:  crystallographic information; 3D view; checkCIF report

## Figures and Tables

**Table 1:** Hydrogen-bond geometry (Å, °)

*D*—H⋯*A*	*D*—H	H⋯*A*	*D*⋯*A*	*D*—H⋯*A*
N3—H3⋯N2	0.86	2.18	2.588 (2)	109
N6—H6⋯N5	0.86	2.18	2.585 (2)	109
C1—H1*B*⋯O2	0.96	2.45	2.921 (3)	110
C2—H2*B*⋯O1	0.96	2.46	2.866 (3)	105

**Fig. 1.**
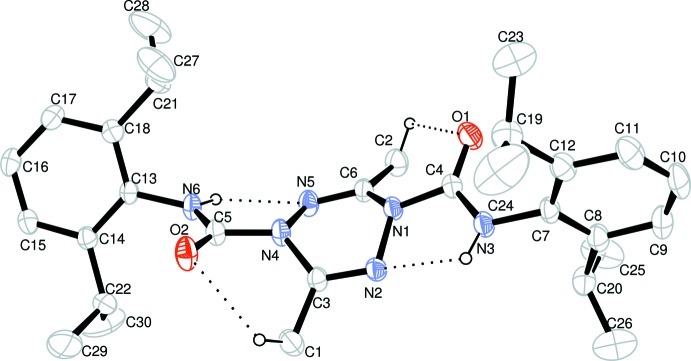
The molecular structure of (I), shown with 30% probability displacement ellipsoids. Hydrogen bonds are shown as dashed lines.

## supplementary crystallographic information

### Comment

Tetrazine derivatives have high activity in chemical reactions (Domingo *et al.*, 2009; Lorincz *et al.*, 2010), and have been widely used in pesticides and medicines (Devaraj *et al.*, 2009; Eremeev *et al.*,1978, 1980; Han *et al.*, 2010; Neunhoeffer, 1984; Sauer, 1996). In a continuation of our studies of antitumor activities in 1,2,4,5-tetrazine derivatives (Hu *et al.*, 2002, 2004; Rao & Hu, 2005, 2006), we have obtained a colourless crystalline compound, (I). The structure was confirmed by single-crystal X-ray diffraction.

The molecular structure of (I) is illustrated in Fig. 1. The N2═C3 [1.274 (2) Å] and N5═C6 [1.270 (2) Å] bonds are typical double bonds, and the C3—N4 [1.403 (2) Å], the N4—N5 [1.423 (2) Å], C6—N1 [1.405 (2) Å] and N1—N2 [1.424 (2) Å] bond lengths correspond to typical single bonds (Allen *et al.*, 1987). The tetrazine ring is a 1,4-dihydro structure with the N-substituted groups at the 1,4-positions.

In (I), atoms N2, C3, N5 and C6 are approximately planar, with the largest deviation from this plane being 0.025 (1) Å. Atoms N1 and N4 deviate from this plane by -0.457 (3) and -0.463 (3) Å, respectively. The dihedral angle between the N2/C3/N5/C6 plane and the N1/N2/C6 plane is 36.75 (21)°, and between the N2/C3/N5/C6 plane and the N4/N5/C3 plane is 37.24 (12)°. The tetrazine ring has a boat conformation. The dihedral angles between the N2/C3/N5/C6 plane and the two benzene rings are 85.42 (9) and 47.28 (8)°, respectively. And the two benzene rings form a dihedral angle of 47.69 (9)°. Intramolecular N—H···N and weak C—H···O hydrogen bonds are observed.

### Experimental

The title compound was the product of the reaction of 3,6-dimethyl-1,6-dihydro-1,2,4,5-tetrazine, bis(trichloromethyl) carbonate and 2,6-diisopropylaniline according to the procedure (Hu *et al.*, 2004; Rao *et al.*, 2012; Skorianetz & Kovats, 1970, 1971; Sun *et al.*, 2003). A solution of the compound in ethanol was concentrated gradually at room temperature to afford colourless blocks.

### Refinement

H atoms were included in calculated positions and refined using a riding model. H atoms were given isotropic displacement parameters equal to 1.2 (or 1.5 for methyl H atoms) times the equivalent isotropic displacement parameters of their parent atoms, and C—H distances were set to 0.96 Å for methyl H atoms, 0.93 Å for phenyl H atoms and 0.98 Å for methine H atoms, while N—H distances were set to 0.86 Å.

### Crystal data

**Table:**

C_30_H_42_N_6_O_2_	*F*(000) = 1120
*M**_r_* = 518.70	*D*_x_ = 1.143 Mg m^−^^3^
Monoclinic, *P*2_1_/*n*	Mo *K*α radiation, λ = 0.71073 Å
Hall symbol: -P 2yn	Cell parameters from 9937 reflections
*a* = 9.0599 (14) Å	θ = 2.4–22.7°
*b* = 33.203 (5) Å	µ = 0.07 mm^−^^1^
*c* = 10.8082 (17) Å	*T* = 298 K
β = 112.013 (2)°	Block, colourless
*V* = 3014.3 (8) Å^3^	0.38 × 0.28 × 0.20 mm
*Z* = 4	

### Data collection

**Table:**

Bruker SMART CCD diffractometer	5304 independent reflections
Radiation source: fine-focus sealed tube	3918 reflections with *I* > 2σ(*I*)
Graphite monochromator	*R*_int_ = 0.029
φ and ω scans	θ_max_ = 25.0°, θ_min_ = 2.1°
Absorption correction: multi-scan (*SADABS*; Bruker, 1997)	*h* = −9→10
*T*_min_ = 0.973, *T*_max_ = 0.986	*k* = −39→26
15158 measured reflections	*l* = −12→10

### Refinement

**Table:**

Refinement on *F*^2^	Primary atom site location: structure-invariant direct methods
Least-squares matrix: full	Secondary atom site location: difference Fourier map
*R*[*F*^2^ > 2σ(*F*^2^)] = 0.057	Hydrogen site location: inferred from neighbouring sites
*wR*(*F*^2^) = 0.154	H-atom parameters constrained
*S* = 1.05	*w* = 1/[σ^2^(*F*_o_^2^) + (0.0755*P*)^2^ + 0.6253*P*] where *P* = (*F*_o_^2^ + 2*F*_c_^2^)/3
5304 reflections	(Δ/σ)_max_ < 0.001
352 parameters	Δρ_max_ = 0.20 e Å^−^^3^
0 restraints	Δρ_min_ = −0.22 e Å^−^^3^

### Special details

**Table:**

Geometry. All esds (except the esd in the dihedral angle between two l.s. planes) are estimated using the full covariance matrix. The cell esds are taken into account individually in the estimation of esds in distances, angles and torsion angles; correlations between esds in cell parameters are only used when they are defined by crystal symmetry. An approximate (isotropic) treatment of cell esds is used for estimating esds involving l.s. planes.
Refinement. Refinement of F^2^ against ALL reflections. The weighted R-factor wR and goodness of fit S are based on F^2^, conventional R-factors R are based on F, with F set to zero for negative F^2^. The threshold expression of F^2^ > 2sigma(F^2^) is used only for calculating R-factors(gt) etc. and is not relevant to the choice of reflections for refinement. R-factors based on F^2^ are statistically about twice as large as those based on F, and R- factors based on ALL data will be even larger.

### Fractional atomic coordinates and isotropic or equivalent isotropic displacement parameters (Å^2^)

**Table:**

	*x*	*y*	*z*	*U*_iso_*/*U*_eq_	
O1	0.4285 (2)	0.09149 (5)	0.40846 (15)	0.0630 (5)	
O2	0.43830 (19)	0.24162 (4)	0.01245 (15)	0.0601 (5)	
N1	0.4915 (2)	0.12554 (5)	0.25237 (15)	0.0409 (4)	
N2	0.4850 (2)	0.12384 (5)	0.11873 (15)	0.0423 (4)	
N3	0.3522 (2)	0.06577 (5)	0.19903 (17)	0.0458 (4)	
H3	0.3448	0.0704	0.1186	0.055*	
N4	0.5403 (2)	0.19120 (5)	0.16262 (15)	0.0444 (4)	
N5	0.6465 (2)	0.18257 (5)	0.29506 (16)	0.0448 (4)	
N6	0.6382 (2)	0.25574 (5)	0.21099 (16)	0.0435 (4)	
H6	0.7105	0.2450	0.2793	0.052*	
C1	0.5118 (3)	0.16145 (7)	−0.0599 (2)	0.0554 (6)	
H1A	0.6140	0.1715	−0.0529	0.083*	
H1B	0.4301	0.1796	−0.1130	0.083*	
H1C	0.4947	0.1354	−0.1013	0.083*	
C2	0.7190 (3)	0.13600 (7)	0.4748 (2)	0.0549 (6)	
H2A	0.7613	0.1098	0.4696	0.082*	
H2B	0.6580	0.1347	0.5306	0.082*	
H2C	0.8050	0.1548	0.5121	0.082*	
C3	0.5067 (2)	0.15814 (6)	0.07548 (19)	0.0394 (5)	
C4	0.4247 (2)	0.09282 (6)	0.2953 (2)	0.0430 (5)	
C5	0.5345 (2)	0.23148 (6)	0.1203 (2)	0.0435 (5)	
C6	0.6150 (2)	0.14950 (6)	0.33887 (19)	0.0403 (5)	
C7	0.2869 (3)	0.02908 (6)	0.2285 (2)	0.0441 (5)	
C8	0.3805 (3)	−0.00559 (6)	0.2557 (2)	0.0506 (6)	
C9	0.3178 (3)	−0.04044 (7)	0.2882 (2)	0.0628 (7)	
H9	0.3786	−0.0639	0.3084	0.075*	
C10	0.1687 (3)	−0.04073 (8)	0.2906 (3)	0.0681 (7)	
H10	0.1285	−0.0644	0.3121	0.082*	
C11	0.0774 (3)	−0.00655 (8)	0.2619 (2)	0.0650 (7)	
H11	−0.0245	−0.0075	0.2635	0.078*	
C12	0.1332 (3)	0.02956 (7)	0.2302 (2)	0.0519 (6)	
C13	0.6338 (2)	0.29878 (6)	0.19910 (19)	0.0376 (5)	
C14	0.6868 (2)	0.31737 (6)	0.10760 (19)	0.0407 (5)	
C15	0.6880 (3)	0.35907 (6)	0.1049 (2)	0.0480 (5)	
H15	0.7230	0.3722	0.0449	0.058*	
C16	0.6386 (3)	0.38145 (6)	0.1892 (2)	0.0518 (6)	
H16	0.6401	0.4094	0.1857	0.062*	
C17	0.5867 (3)	0.36249 (7)	0.2788 (2)	0.0516 (6)	
H17	0.5540	0.3779	0.3358	0.062*	
C18	0.5826 (2)	0.32085 (6)	0.2854 (2)	0.0433 (5)	
C19	0.0314 (3)	0.06713 (8)	0.1971 (3)	0.0684 (7)	
H19	0.1005	0.0899	0.1982	0.082*	
C20	0.5466 (3)	−0.00640 (7)	0.2537 (2)	0.0585 (6)	
H20	0.5678	0.0203	0.2253	0.070*	
C21	0.5165 (3)	0.30030 (8)	0.3790 (2)	0.0559 (6)	
H21	0.5600	0.2729	0.3944	0.067*	
C22	0.7482 (3)	0.29395 (7)	0.0170 (2)	0.0551 (6)	
H22	0.7259	0.2654	0.0244	0.066*	
C23	−0.0464 (5)	0.07606 (12)	0.2968 (4)	0.1242 (14)	
H23A	−0.0918	0.1026	0.2809	0.186*	
H23B	0.0322	0.0746	0.3858	0.186*	
H23C	−0.1287	0.0567	0.2865	0.186*	
C24	−0.0932 (6)	0.06421 (15)	0.0601 (4)	0.161 (2)	
H24A	−0.1517	0.0890	0.0380	0.241*	
H24B	−0.1645	0.0425	0.0569	0.241*	
H24C	−0.0435	0.0592	−0.0028	0.241*	
C25	0.6701 (4)	−0.01415 (13)	0.3899 (3)	0.1023 (12)	
H25A	0.7742	−0.0130	0.3860	0.153*	
H25B	0.6535	−0.0403	0.4200	0.153*	
H25C	0.6619	0.0059	0.4509	0.153*	
C26	0.5627 (4)	−0.03696 (12)	0.1547 (3)	0.1049 (12)	
H26A	0.6654	−0.0342	0.1490	0.157*	
H26B	0.4812	−0.0323	0.0685	0.157*	
H26C	0.5516	−0.0637	0.1842	0.157*	
C27	0.3390 (3)	0.29666 (12)	0.3105 (3)	0.0984 (11)	
H27A	0.2963	0.2835	0.3689	0.148*	
H27B	0.3142	0.2812	0.2303	0.148*	
H27C	0.2931	0.3230	0.2886	0.148*	
C28	0.5620 (5)	0.32021 (13)	0.5130 (3)	0.1210 (14)	
H28A	0.5167	0.3055	0.5666	0.182*	
H28B	0.5226	0.3473	0.5015	0.182*	
H28C	0.6758	0.3205	0.5566	0.182*	
C29	0.6639 (6)	0.30637 (12)	−0.1280 (3)	0.1413 (19)	
H29A	0.7050	0.2910	−0.1832	0.212*	
H29B	0.6818	0.3345	−0.1374	0.212*	
H29C	0.5518	0.3015	−0.1551	0.212*	
C30	0.9261 (4)	0.29894 (13)	0.0624 (4)	0.1227 (15)	
H30A	0.9769	0.2860	0.1472	0.184*	
H30B	0.9522	0.3271	0.0708	0.184*	
H30C	0.9624	0.2869	−0.0019	0.184*	

### Atomic displacement parameters (Å^2^)

**Table:**

	*U*^11^	*U*^22^	*U*^33^	*U*^12^	*U*^13^	*U*^23^
O1	0.0907 (13)	0.0552 (10)	0.0486 (10)	−0.0230 (9)	0.0324 (9)	−0.0020 (7)
O2	0.0637 (10)	0.0441 (9)	0.0530 (10)	−0.0064 (7)	−0.0004 (8)	0.0117 (7)
N1	0.0535 (10)	0.0331 (9)	0.0360 (9)	−0.0093 (8)	0.0167 (8)	0.0007 (7)
N2	0.0519 (10)	0.0366 (10)	0.0377 (9)	−0.0061 (8)	0.0160 (8)	−0.0002 (7)
N3	0.0583 (11)	0.0367 (10)	0.0427 (10)	−0.0117 (8)	0.0194 (8)	0.0012 (8)
N4	0.0533 (10)	0.0331 (9)	0.0368 (9)	−0.0080 (8)	0.0056 (8)	0.0046 (7)
N5	0.0497 (10)	0.0364 (10)	0.0389 (9)	−0.0066 (8)	0.0058 (8)	0.0025 (7)
N6	0.0484 (10)	0.0311 (9)	0.0439 (10)	−0.0047 (8)	0.0092 (8)	0.0049 (7)
C1	0.0665 (15)	0.0546 (14)	0.0455 (12)	−0.0111 (12)	0.0216 (11)	0.0033 (10)
C2	0.0591 (14)	0.0503 (13)	0.0451 (13)	−0.0012 (11)	0.0079 (10)	0.0064 (10)
C3	0.0403 (11)	0.0351 (11)	0.0401 (11)	−0.0057 (9)	0.0119 (9)	0.0019 (9)
C4	0.0500 (12)	0.0354 (11)	0.0453 (12)	−0.0022 (9)	0.0197 (10)	0.0041 (9)
C5	0.0455 (12)	0.0361 (11)	0.0447 (12)	−0.0050 (9)	0.0122 (10)	0.0053 (9)
C6	0.0453 (11)	0.0333 (11)	0.0397 (11)	−0.0028 (9)	0.0130 (9)	0.0014 (9)
C7	0.0515 (13)	0.0359 (11)	0.0423 (11)	−0.0139 (10)	0.0146 (10)	−0.0008 (9)
C8	0.0575 (14)	0.0415 (12)	0.0492 (13)	−0.0113 (11)	0.0159 (10)	0.0008 (10)
C9	0.0733 (18)	0.0395 (13)	0.0695 (16)	−0.0113 (12)	0.0198 (13)	0.0071 (11)
C10	0.0767 (19)	0.0516 (16)	0.0702 (17)	−0.0283 (14)	0.0210 (14)	0.0079 (12)
C11	0.0538 (14)	0.0732 (18)	0.0658 (16)	−0.0249 (14)	0.0200 (12)	0.0025 (13)
C12	0.0498 (13)	0.0547 (14)	0.0485 (13)	−0.0134 (11)	0.0151 (10)	−0.0016 (10)
C13	0.0372 (10)	0.0303 (10)	0.0420 (11)	−0.0039 (8)	0.0109 (9)	0.0016 (8)
C14	0.0436 (11)	0.0369 (11)	0.0405 (11)	−0.0039 (9)	0.0145 (9)	0.0018 (9)
C15	0.0536 (13)	0.0393 (12)	0.0529 (13)	−0.0037 (10)	0.0220 (10)	0.0080 (10)
C16	0.0532 (13)	0.0313 (11)	0.0684 (15)	−0.0022 (10)	0.0201 (11)	0.0027 (10)
C17	0.0533 (13)	0.0421 (13)	0.0603 (14)	0.0061 (10)	0.0225 (11)	−0.0027 (11)
C18	0.0370 (11)	0.0458 (12)	0.0453 (12)	0.0015 (9)	0.0133 (9)	0.0042 (9)
C19	0.0544 (15)	0.0691 (17)	0.0824 (19)	−0.0005 (13)	0.0263 (13)	0.0042 (14)
C20	0.0624 (15)	0.0440 (13)	0.0693 (16)	−0.0008 (11)	0.0247 (12)	0.0060 (11)
C21	0.0591 (14)	0.0615 (15)	0.0561 (14)	0.0047 (11)	0.0320 (12)	0.0100 (11)
C22	0.0736 (16)	0.0491 (13)	0.0507 (13)	−0.0040 (12)	0.0324 (12)	−0.0005 (10)
C23	0.149 (4)	0.111 (3)	0.135 (3)	0.018 (3)	0.079 (3)	−0.018 (2)
C24	0.176 (4)	0.157 (4)	0.087 (3)	0.081 (4)	−0.022 (3)	−0.010 (3)
C25	0.0665 (19)	0.168 (4)	0.067 (2)	−0.019 (2)	0.0188 (15)	−0.009 (2)
C26	0.085 (2)	0.139 (3)	0.088 (2)	0.010 (2)	0.0287 (18)	−0.029 (2)
C27	0.0706 (19)	0.142 (3)	0.094 (2)	−0.0177 (19)	0.0439 (17)	0.028 (2)
C28	0.150 (3)	0.166 (4)	0.0577 (19)	−0.048 (3)	0.052 (2)	−0.009 (2)
C29	0.260 (6)	0.115 (3)	0.0521 (18)	0.059 (3)	0.063 (3)	0.0101 (19)
C30	0.090 (2)	0.136 (3)	0.176 (4)	−0.023 (2)	0.089 (3)	−0.070 (3)

### Geometric parameters (Å, º)

**Table:**

O1—C4	1.211 (2)	C16—C17	1.377 (3)
O2—C5	1.212 (2)	C16—H16	0.9300
N1—C6	1.405 (2)	C17—C18	1.386 (3)
N1—C4	1.405 (2)	C17—H17	0.9300
N1—N2	1.424 (2)	C18—C21	1.517 (3)
N2—C3	1.274 (2)	C19—C24	1.492 (4)
N3—C4	1.345 (3)	C19—C23	1.521 (4)
N3—C7	1.441 (2)	C19—H19	0.9800
N3—H3	0.8600	C20—C25	1.500 (4)
N4—C3	1.403 (2)	C20—C26	1.521 (4)
N4—C5	1.408 (2)	C20—H20	0.9800
N4—N5	1.423 (2)	C21—C27	1.502 (4)
N5—C6	1.270 (2)	C21—C28	1.502 (4)
N6—C5	1.342 (3)	C21—H21	0.9800
N6—C13	1.434 (2)	C22—C30	1.508 (4)
N6—H6	0.8600	C22—C29	1.521 (4)
C1—C3	1.486 (3)	C22—H22	0.9800
C1—H1A	0.9600	C23—H23A	0.9600
C1—H1B	0.9600	C23—H23B	0.9600
C1—H1C	0.9600	C23—H23C	0.9600
C2—C6	1.487 (3)	C24—H24A	0.9600
C2—H2A	0.9600	C24—H24B	0.9600
C2—H2B	0.9600	C24—H24C	0.9600
C2—H2C	0.9600	C25—H25A	0.9600
C7—C8	1.394 (3)	C25—H25B	0.9600
C7—C12	1.400 (3)	C25—H25C	0.9600
C8—C9	1.390 (3)	C26—H26A	0.9600
C8—C20	1.514 (3)	C26—H26B	0.9600
C9—C10	1.361 (4)	C26—H26C	0.9600
C9—H9	0.9300	C27—H27A	0.9600
C10—C11	1.370 (4)	C27—H27B	0.9600
C10—H10	0.9300	C27—H27C	0.9600
C11—C12	1.393 (3)	C28—H28A	0.9600
C11—H11	0.9300	C28—H28B	0.9600
C12—C19	1.512 (3)	C28—H28C	0.9600
C13—C14	1.395 (3)	C29—H29A	0.9600
C13—C18	1.395 (3)	C29—H29B	0.9600
C14—C15	1.385 (3)	C29—H29C	0.9600
C14—C22	1.511 (3)	C30—H30A	0.9600
C15—C16	1.374 (3)	C30—H30B	0.9600
C15—H15	0.9300	C30—H30C	0.9600
			
C6—N1—C4	123.52 (16)	C13—C18—C21	121.51 (19)
C6—N1—N2	114.68 (15)	C24—C19—C12	110.4 (2)
C4—N1—N2	116.42 (15)	C24—C19—C23	109.7 (3)
C3—N2—N1	112.52 (16)	C12—C19—C23	113.4 (3)
C4—N3—C7	121.03 (17)	C24—C19—H19	107.7
C4—N3—H3	119.5	C12—C19—H19	107.7
C7—N3—H3	119.5	C23—C19—H19	107.7
C3—N4—C5	123.54 (16)	C25—C20—C8	111.7 (2)
C3—N4—N5	114.38 (16)	C25—C20—C26	110.1 (3)
C5—N4—N5	116.55 (15)	C8—C20—C26	112.3 (2)
C6—N5—N4	112.80 (16)	C25—C20—H20	107.5
C5—N6—C13	122.97 (16)	C8—C20—H20	107.5
C5—N6—H6	118.5	C26—C20—H20	107.5
C13—N6—H6	118.5	C27—C21—C28	111.5 (3)
C3—C1—H1A	109.5	C27—C21—C18	108.96 (19)
C3—C1—H1B	109.5	C28—C21—C18	114.2 (2)
H1A—C1—H1B	109.5	C27—C21—H21	107.3
C3—C1—H1C	109.5	C28—C21—H21	107.3
H1A—C1—H1C	109.5	C18—C21—H21	107.3
H1B—C1—H1C	109.5	C30—C22—C14	109.9 (2)
C6—C2—H2A	109.5	C30—C22—C29	111.6 (3)
C6—C2—H2B	109.5	C14—C22—C29	111.3 (2)
H2A—C2—H2B	109.5	C30—C22—H22	107.9
C6—C2—H2C	109.5	C14—C22—H22	107.9
H2A—C2—H2C	109.5	C29—C22—H22	107.9
H2B—C2—H2C	109.5	C19—C23—H23A	109.5
N2—C3—N4	118.53 (17)	C19—C23—H23B	109.5
N2—C3—C1	119.58 (18)	H23A—C23—H23B	109.5
N4—C3—C1	121.60 (17)	C19—C23—H23C	109.5
O1—C4—N3	125.31 (19)	H23A—C23—H23C	109.5
O1—C4—N1	120.60 (18)	H23B—C23—H23C	109.5
N3—C4—N1	114.00 (18)	C19—C24—H24A	109.5
O2—C5—N6	126.14 (19)	C19—C24—H24B	109.5
O2—C5—N4	120.12 (18)	H24A—C24—H24B	109.5
N6—C5—N4	113.69 (17)	C19—C24—H24C	109.5
N5—C6—N1	118.39 (17)	H24A—C24—H24C	109.5
N5—C6—C2	118.78 (18)	H24B—C24—H24C	109.5
N1—C6—C2	122.60 (17)	C20—C25—H25A	109.5
C8—C7—C12	122.39 (19)	C20—C25—H25B	109.5
C8—C7—N3	118.50 (19)	H25A—C25—H25B	109.5
C12—C7—N3	119.11 (19)	C20—C25—H25C	109.5
C9—C8—C7	117.9 (2)	H25A—C25—H25C	109.5
C9—C8—C20	119.4 (2)	H25B—C25—H25C	109.5
C7—C8—C20	122.74 (19)	C20—C26—H26A	109.5
C10—C9—C8	120.9 (2)	C20—C26—H26B	109.5
C10—C9—H9	119.6	H26A—C26—H26B	109.5
C8—C9—H9	119.6	C20—C26—H26C	109.5
C9—C10—C11	120.6 (2)	H26A—C26—H26C	109.5
C9—C10—H10	119.7	H26B—C26—H26C	109.5
C11—C10—H10	119.7	C21—C27—H27A	109.5
C10—C11—C12	121.7 (2)	C21—C27—H27B	109.5
C10—C11—H11	119.2	H27A—C27—H27B	109.5
C12—C11—H11	119.2	C21—C27—H27C	109.5
C11—C12—C7	116.6 (2)	H27A—C27—H27C	109.5
C11—C12—C19	121.5 (2)	H27B—C27—H27C	109.5
C7—C12—C19	121.8 (2)	C21—C28—H28A	109.5
C14—C13—C18	122.05 (18)	C21—C28—H28B	109.5
C14—C13—N6	119.95 (17)	H28A—C28—H28B	109.5
C18—C13—N6	117.94 (17)	C21—C28—H28C	109.5
C15—C14—C13	117.70 (19)	H28A—C28—H28C	109.5
C15—C14—C22	119.55 (18)	H28B—C28—H28C	109.5
C13—C14—C22	122.70 (18)	C22—C29—H29A	109.5
C16—C15—C14	121.3 (2)	C22—C29—H29B	109.5
C16—C15—H15	119.3	H29A—C29—H29B	109.5
C14—C15—H15	119.3	C22—C29—H29C	109.5
C15—C16—C17	120.0 (2)	H29A—C29—H29C	109.5
C15—C16—H16	120.0	H29B—C29—H29C	109.5
C17—C16—H16	120.0	C22—C30—H30A	109.5
C16—C17—C18	121.0 (2)	C22—C30—H30B	109.5
C16—C17—H17	119.5	H30A—C30—H30B	109.5
C18—C17—H17	119.5	C22—C30—H30C	109.5
C17—C18—C13	117.86 (19)	H30A—C30—H30C	109.5
C17—C18—C21	120.6 (2)	H30B—C30—H30C	109.5
			
C6—N1—N2—C3	−42.8 (2)	C10—C11—C12—C7	0.4 (3)
C4—N1—N2—C3	162.07 (18)	C10—C11—C12—C19	179.4 (2)
C3—N4—N5—C6	−43.5 (2)	C8—C7—C12—C11	0.5 (3)
C5—N4—N5—C6	161.71 (19)	N3—C7—C12—C11	−178.75 (19)
N1—N2—C3—N4	2.9 (3)	C8—C7—C12—C19	−178.5 (2)
N1—N2—C3—C1	176.89 (17)	N3—C7—C12—C19	2.3 (3)
C5—N4—C3—N2	−166.59 (19)	C5—N6—C13—C14	−74.7 (3)
N5—N4—C3—N2	40.6 (3)	C5—N6—C13—C18	108.2 (2)
C5—N4—C3—C1	19.6 (3)	C18—C13—C14—C15	0.3 (3)
N5—N4—C3—C1	−133.2 (2)	N6—C13—C14—C15	−176.64 (17)
C7—N3—C4—O1	−7.7 (3)	C18—C13—C14—C22	177.70 (19)
C7—N3—C4—N1	175.78 (18)	N6—C13—C14—C22	0.8 (3)
C6—N1—C4—O1	25.7 (3)	C13—C14—C15—C16	−0.1 (3)
N2—N1—C4—O1	178.37 (19)	C22—C14—C15—C16	−177.6 (2)
C6—N1—C4—N3	−157.65 (18)	C14—C15—C16—C17	0.1 (3)
N2—N1—C4—N3	−5.0 (3)	C15—C16—C17—C18	−0.3 (3)
C13—N6—C5—O2	8.8 (3)	C16—C17—C18—C13	0.5 (3)
C13—N6—C5—N4	−168.69 (17)	C16—C17—C18—C21	−176.5 (2)
C3—N4—C5—O2	33.3 (3)	C14—C13—C18—C17	−0.5 (3)
N5—N4—C5—O2	−174.45 (19)	N6—C13—C18—C17	176.52 (18)
C3—N4—C5—N6	−149.00 (19)	C14—C13—C18—C21	176.49 (18)
N5—N4—C5—N6	3.2 (3)	N6—C13—C18—C21	−6.5 (3)
N4—N5—C6—N1	3.7 (3)	C11—C12—C19—C24	−75.3 (4)
N4—N5—C6—C2	178.32 (18)	C7—C12—C19—C24	103.7 (3)
C4—N1—C6—N5	−166.90 (19)	C11—C12—C19—C23	48.3 (4)
N2—N1—C6—N5	40.0 (3)	C7—C12—C19—C23	−132.8 (3)
C4—N1—C6—C2	18.7 (3)	C9—C8—C20—C25	−64.7 (3)
N2—N1—C6—C2	−134.4 (2)	C7—C8—C20—C25	114.2 (3)
C4—N3—C7—C8	−94.6 (2)	C9—C8—C20—C26	59.5 (3)
C4—N3—C7—C12	84.7 (2)	C7—C8—C20—C26	−121.6 (3)
C12—C7—C8—C9	−1.3 (3)	C17—C18—C21—C27	85.4 (3)
N3—C7—C8—C9	177.98 (19)	C13—C18—C21—C27	−91.5 (3)
C12—C7—C8—C20	179.8 (2)	C17—C18—C21—C28	−40.1 (3)
N3—C7—C8—C20	−0.9 (3)	C13—C18—C21—C28	143.1 (3)
C7—C8—C9—C10	1.2 (3)	C15—C14—C22—C30	69.4 (3)
C20—C8—C9—C10	−179.9 (2)	C13—C14—C22—C30	−107.9 (3)
C8—C9—C10—C11	−0.3 (4)	C15—C14—C22—C29	−54.8 (3)
C9—C10—C11—C12	−0.5 (4)	C13—C14—C22—C29	127.8 (3)

### Hydrogen-bond geometry (Å, º)

**Table:**

*D*—H···*A*	*D*—H	H···*A*	*D*···*A*	*D*—H···*A*
N3—H3···N2	0.86	2.18	2.588 (2)	109
N6—H6···N5	0.86	2.18	2.585 (2)	109
C1—H1*B*···O2	0.96	2.45	2.921 (3)	110
C2—H2*B*···O1	0.96	2.46	2.866 (3)	105
